# Adults Aging With Spinal Cord Injury: Prevalence and Associated Risk Factors for Diagnosis of Diabetes Mellitus

**DOI:** 10.1093/geroni/igab046.3175

**Published:** 2021-12-17

**Authors:** Seeun Park, Lisa Bratzke

**Affiliations:** University of Wisconsin-Madison, Madison, Wisconsin, United States; University of Wisconsin-Madison, Madison, Wisconsin, United States

## Abstract

With the increased life expectancy, people aging with spinal cord injury (SCI) are more likely to experience chronic conditions, including diabetes mellitus (DM). The results of previous literature related to the prevalence of DM are mixed and risk factors associated with diagnosis of DM after SCI are not well defined. This study aims to investigate the prevalence of DM and explore associated risk factors for diagnosis of DM among adults aging with long-standing spinal cord injury in the United States. This is a secondary data analysis using the National Spinal Cord Injury Model Systems Database. Participants included 516 people age 45 and older who have been living with SCI for more than 10 years. The prevalence of DM in this sample was 13.2%. Multivariate logistic regression, controlling for confounding variables, was conducted to identify risk factors associated with DM diagnosis in this sample. The multivariate logistic regression model found that the participants who responded with less severe SCI measured by the ASIA impairment scale were less likely to be diagnosed with DM (OR=0.332, p=.017). Also, DM was found to be significantly associated with BMI (OR=1.043, p=.010) and age (OR=1.038, p= .010) respectively. Duration of disability was not significantly associated with DM. Future research is needed to validate these findings and identify other common risk factors for DM such as diet/nutrition. Further, exploration of the effect size of risk factors is also warranted. Such findings will inform interventions to aid prevention and early detection of DM.

